# Dental health: EPMA recommendations for innovative strategies

**DOI:** 10.1186/1878-5085-5-S1-A119

**Published:** 2014-02-11

**Authors:** Olga Golubnitschaja, Vincenzo Costigliola

**Affiliations:** 1 Molecular Diagnostics, Radiological Clinic, Medical Faculty, Friedrich-Wilhelms-University of Bonn, Germany; 2 The European Association for Predictive, Preventive and Personalised Medicine, Brussels, Belgium

## Scientific background

Oral/dental health contributes to the overall health and well being of everybody. A growing body of evidence demonstrates that the manifested dental and oral pathologies are linked to the increased risk of various diseases including heart and lung disease, vascular pathologies, stroke, diabetes mellitus (type 1, 2 and 3), neurological disorders, pre-term birth, and even some types of cancer, among others. Further, certain oral symptoms are considered as the early indicator of a spectrum of the mental disorders such as anorexia, bulimia, anxiety and depression. On the other hand, dental diseases themselves may be caused by acute and chronic systemic disorders such as diabetes mellitus.

While an association between oral/dental diseases and systemic disorders is well-established, the cause-and-effect relationships in these conditions are poorly understood. An oral infection and chronic inflammation constitute the basis for currently popular theory of the functional link between dental and other pathologies. However, it is unlikely that this theory alone explains the whole spectrum of clinical situations often attributed to the oral-systemic connection. Thus, investigation of the cause-and-effect relationship of these conditions is a prerequisite for predictive, preventive and personalised dental medicine (PPPDM).

## Recommendations

Oral/dental health is related to the entire body which is also impacted by cumulative effects of systemic disorders. Consequently, in dentistry currently applied reactive medical approaches should be reconsidered in favor of predictive diagnostics, targeted prevention and treatments tailored to the person. To that end following measures are recommended:

➣ Propagation of multidisciplinary expertise in dentistry, in order to shift the paradigm from reactive medical services to PPPDM

➣ Networking of scientific and dental/medical centres for PPPDM

➣ Promotion of specialised educational programmes for professionals and the general population

➣ Creation of multidisciplinary scientific projects (“Horizon 2020” and other international programmes) dedicated to the cause-and-effect relationship between predisposition to, onset and progression of dental diseases and abnormalities of other organs

➣ Consideration of both genetic and epigenetic risk factors for dental disorders

➣ Promotion of the high quality biomarker research in dental disorders with consequent clinical implementation for diagnostic and treatment purposes

➣ Development of integrative bioinformatics in dentistry: disease modelling, patient profiling, complementary levels of analytical technologies, individualised algorithms for tailored treatments, etc.

➣ Inclusion of advanced dental services into the clustered PPPDM centres: the aspects of healthcare economy and issue relevant legislation should be thoroughly considered.

For the practical realisation of the above listed objectives in a global scale, EPMA has created the specialised section: “Department of Predictive, Preventive and Personalised Medicine”, DPPPD, http://www.epmanet.eu/index.php/component/content/article/109-dpppd

The EPMA-DPPPD is EU unrestricted and is supported by the thematic section in the EPMA Journal (BMC, PubMed-indexed “open access” journal) - http://www.epmajournal.com/

